# Epidemiology of Confirmed COVID-19 Deaths in Adults, England, March–December 2020

**DOI:** 10.3201/eid2705.203524

**Published:** 2021-05

**Authors:** Alison E. Brown, Ellen Heinsbroek, Meaghan M. Kall, Hester Allen, Kazim Beebeejaun, Paula Blomquist, Ines Campos-Matos, Colin N.J. Campbell, Hamish Mohammed, Katy Sinka, Theresa Lamagni, Nicholas Phin, the PHE COVID-19 Mortality Working Group,, Gavin Dabrera

**Affiliations:** Public Health England, London, UK

**Keywords:** COVID-19, coronavirus disease, SARS-CoV-2, severe acute respiratory syndrome coronavirus 2, viruses, respiratory infections, zoonoses, England, United Kingdom

## Abstract

Of the 58,186 coronavirus deaths among adults in England during March–December 2020, 77% occurred in hospitals, 93% were in patients >60 years, and 91% occurred within 28 days of positive specimen. Cumulative mortality rates were highest among persons of Black, Asian, other, or mixed ethnicities and in socioeconomically deprived areas.

Reliable ascertainment and description of mortality rates is vital in monitoring the public health response to coronavirus disease (COVID-19). Disparities between population subgroups provide critical insights into which groups are most affected, directly informing the public response. In England, COVID-19 was first detected on January 30, 2020; the first COVID-19 death occurred on March 2, 2020. We describe trends in COVID-19 mortality rates by age group, sex, ethnicity, residential region and socioeconomic deprivation, time from positive specimen date to death, and place of death during the first 9 months after the first known COVID-19 death in England.

## The Study

Public Health England (PHE) receives daily reports of the date of death, the date the specimen is taken for COVID-19 testing, and laboratory results for adults >18 years of age from 3 sources: hospital trusts using the COVID-19 Patient Notification System; local PHE Health Protection Teams (for nonhospital settings); and the National Health Service Demographic Batch Service, which matches of all laboratory-confirmed COVID-19 cases against registered deaths records. Data from each source were combined daily into a single dataset, the COVID-19 Specific Mortality Surveillance System (COSMOSS) (*1*).

COSMOSS data are matched using a unique patient identifier (National Health Service number) to death registrations from the Office for National Statistics (*2*) to ascertain setting and cause of death. The data are then matched to Hospital Episode Statistics to identify ethnicity and to area-level data to categorize relative socioeconomic deprivation based on Indices for Multiple Deprivation (IMD) (*3*). Data are deduplicated daily so that 1 record reported from the multiple data sources is retained for each decedent.

COSMOSS COVID-19 deaths are defined as any death occurring within 60 days of a date on which a positive specimen was taken for COVID-19, or any death for which COVID-19 is listed on the death registration (codes U0.71 or U0.72 from the International Classification of Diseases, 10th Revision, Clinical Modification) (*2*). Cumulative mortality rates (deaths/100,000 population) were calculated using denominator data from Office for National Statistics population estimates (*4*).

By December 3, 2020, a total of 58,186 COVID-19 deaths in adults had been reported to PHE, yielding a crude mortality rate of 132 deaths/100,000 population. An additional 31 deaths among children (persons <18 years of age) were reported.

**Figure 1:**
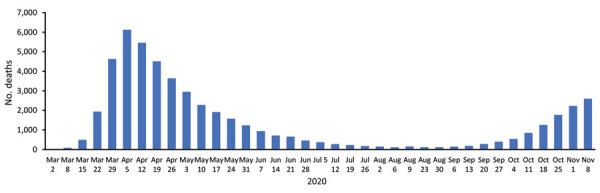
Deaths occurring within 60 days of a laboratory-confirmed coronavirus disease (COVID-19) diagnosis or with COVID-19 on the death registration certificate, by date of death, England, UK, March 2–December 3, 2020.

**Figure 2:**
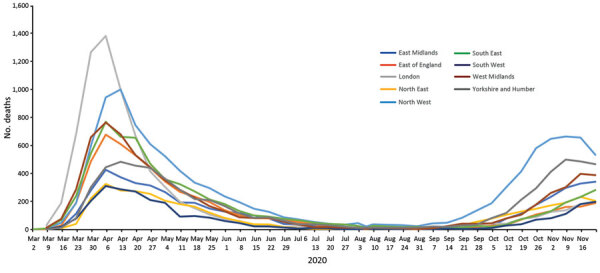
Region-specific coronavirus disease cumulative mortality rate (deaths/100,000 population), by week of death, England, UK, March 2–December 3, 2020.

Social distancing measures were announced nationally on March 23, 2020, 3 weeks after the first death. The number of COVID-19 deaths peaked on April 8, ≈2 weeks after social distancing began (Figure 1, 2), gradually fell to lower levels that were sustained throughout summer, and then increased in late September.

Overall, 93% (54,282) of deaths in adults occurred in persons >60 years of age, and 57% (32,970) occurred in men (Table); 64% of those 18–59 years of age were men. The cumulative mortality rate was 410 deaths/100,000 population among persons >60 years of age, >30 times more than persons 18–59 years of age (13 deaths/100,000 population) (Table). During March 2–December 3, 2020, a total of 0.50% of men and 0.34% of women >60 years of age died, compared with 0.02% of men and 0.01% of women of working age. Irrespective of age, cumulative mortality was highest in men, Black persons, Asian persons, persons of other or mixed ethnic groups, and persons in socioeconomically deprived areas (Table).

**Table:** Coronavirus disease cumulative mortality rates among adults, by age group, sex, region, ethnicity, and residence-based socioeconomic deprivation score, England, UK, March 2–December 3, 2020*

Characteristic	Age group, y						
	18–59				>60		
	No. cumulative deaths	Population†	Cumulative mortality rate§		No. cumulative deaths	Population†	Cumulative mortality rate§
Sex							
F	1,406	15,399,252	9.1		23,809	7,081,746	336
M	2,497	15,399,681	16.2		30,473	6,141,881	496
Region							
London	868	5,266,170	16.5		6,959	1,437,281	484
South East	368	4,554,266	8.1		6,399	2,217,002	289
East of England	371	3,313,330	11.2		5,332	1,646,457	324
West Midlands	479	3,059,310	15.7		6,696	1,405,816	476
Yorkshire and Humber	399	2,854,445	14.0		6,494	1,324,798	490
East Midlands	299	2,486,593	12.0		4,844	1,201,199	403
North West	727	3,803,145	19.1		10,708	1,765,241	607
North East	182	1,377,087	13.2		3,652	686,963	532
South West	156	2,797,918	5.6		2,777	1,568,870	177
Ethnicity							
White	2,625	25,429,023	10.3		48,054	12,384,629	388
Black or Black British	407	1,299,892	31.3		1,523	202,902	751
Asian or Asian British	561	2,920,985	19.2		3,163	510,845	619
Mixed	56	750,086	7.5		241	66,357	363
Other	187	398,948	46.9		866	58,894	1,470
IMD score							
1 (most deprived)	1,299	6,214,331	20.9		13,633	1,934,761	705
2	985	6,414,752	15.4		11,601	2,314,652	501
3	651	5,925,517	11.0		10,226	2,777,687	368
4	537	5,619,071	9.6		9,824	2,965,692	331
5 (least deprived)	377	5,263,233	7.2		8,577	3020639	284
Total*	3,904	30,798,933	12.7		54,282	13,223,627	410

*IMD, Indices for Multiple Deprivation. †Deaths with missing information are excluded: 476 for region, 503 for ethnicity, and 476 for IMD. Population denominator is from 2018 except for IMD. For IMD (*3*), population denominator is 20–59 and >60 year age groups only, and is from 2017. §Deaths/100,000 population.

Place of death was available for 86% (50,227) of decedents. Overall, 77% of deaths occurred in hospitals, 18% in residential care homes, and the remainder elsewhere (e.g., home, hospices, and other communal establishments). The median time from specimen date to death was 8 days (interquartile range 4–15 days); 91% (53,000) died within 28 days of the specimen date. Of those dying in hospital, 96% died within 28 days of the specimen date, compared with 86% in residential care homes and 84% elsewhere.

When we compared first 4.5 month period with the second, the demographic profile of decedents did not change substantially by sex (57% vs. 57% were men), ethnicity (87% vs. 89% White), place of death (77% vs. 78% hospital), or time to death (91% vs. 91% within 28 days of specimen date). However, region of death became less London-focused (Figure 2), and the proportion of deaths occurring in the most deprived residential quintile rose from 24% to 30%.

## Conclusions

In the 9-month period after the first COVID-19 death in England, ≈58,000 adults died of COVID-19 (*5*). COVID-19 deaths disproportionately affected specific adults; 9 of 10 occurred among those >60 years of age. Men, Black persons, Asian persons, persons of other or mixed ethnic groups, and residents in deprived areas also experienced higher cumulative mortality rates compared with White persons and persons in less deprived areas. Almost 1 of 10 persons died >28 days after their specimen date, and 1 of 4 persons died outside hospital.

This comprehensive epidemiologic overview of COVID-19 deaths in England directly informs the pandemic response, including vaccination strategy, and highlights inequalities between populations that require redress (*6*,*7*). Previous reports focused on subsets of deaths, primary-care records, or both. However, our results are consistent with other studies in the United Kingdom (A.B. Docherty et al., unpub. data, https://doi.org/10.1101/2020.04.23.20076042; The OpenSAFELY Collaborative et al., unpub. data, https://doi.org/10.1101/2020.05.06.20092999) that show older age is associated with higher COVID-19 mortality rates (*8*).

The increased rates in older age groups might be attributable to physiologic factors, such as immune senescence (*9*), which, combined with other factors, can increase the risk for acquiring COVID-19 (e.g., frequently receiving healthcare) and complications (e.g., underlying conditions). The higher mortality rates among Black persons, Asian persons, persons of other or mixed ethnic groups, and persons in more deprived areas are probably influenced by factors that reduce capacity to maintain social distancing, including occupation, use of public transportation, crowded or multigenerational housing, and higher rates of chronic conditions (*6*).

The cumulative mortality rate of 132 deaths/100,000 population in our study is consistent with reports from other countries in Western Europe (*10*). However, meaningful comparisons of COVID-19 mortality rates are limited by differing levels of pandemic activity, definitions for COVID-19 deaths, and methods of reporting.

No international standard exists for defining COVID-19 deaths. Some countries exclude deaths occurring >28 days after specimen date; the absence of a time cutoff increases the risk that unrelated deaths are miscoded as COVID-19. Furthermore, daily monitoring of deaths within 28 days might be used as a proxy for underlying incidence and a pragmatic alternative to death registrations that can be delayed. However, we estimate that a 28-day cutoff excludes ≈9% of COVID-19 deaths (*1*). In England, 2 metrics are produced: all deaths within 28 days of specimen date, and all deaths within 60 days of specimen date or with COVID-19 listed on the death certificate (*1*). These definitions were selected after rigorous sensitivity analyses relating to cause of death (*1*). A global consensus on defining COVID-19 deaths is needed urgently.

The PHE mortality reporting system (COSMOSS) was developed rapidly, is comprehensive, and captures deaths daily in all settings (*1*). However, our definition excludes those who died of COVID-19 without having a test and for whom no death registration certificate is yet available. This exclusion is likely most relevant at the start of the epidemic, when tests were only undertaken at hospital admission; we estimate ≈20% of deaths occurred outside of hospitals. Finally, increasing evidence indicates that long-term health problems can occur after COVID-19, but the impact on COVID-19 mortality is unknown.

Further analyses using multivariate models are underway and will better measure the clinical and demographic risk factors for COVID-19 deaths. Furthering understanding of the characteristics of those who die and the context in which they are living is the only way we can reduce COVID-19 mortality overall and address the factors that are driving the inequalities observed across England.
